# A Sex-Specific Analysis of the Predictive Value of Troponin I and T in Patients With and Without Diabetes Mellitus After Successful Coronary Intervention

**DOI:** 10.3389/fendo.2019.00105

**Published:** 2019-03-01

**Authors:** Michael Leutner, Maximilian Tscharre, Serdar Farhan, Hossein Taghizadeh Waghefi, Jürgen Harreiter, Birgit Vogel, Ioannis Tentzeris, Thomas Szekeres, Monika Fritzer-Szekeres, Kurt Huber, Alexandra Kautzky-Willer

**Affiliations:** ^1^Department of Internal Medicine III, Clinical Division of Endocrinology and Metabolism, Unit of Gender Medicine, Medical University of Vienna, Vienna, Austria; ^2^3rd Medical Department, Cardiology and Intensive Care Medicine, Wilhelminenhospital, Vienna, Austria; ^3^Institute for Cardiometabolic Diseases, Karl Landsteiner Society, St. Poelten, Austria; ^4^Icahn School of Medicine at Mount Sinai, The Zena and Michael A. Wiener Cardiovascular Institute, New York, NY, United States; ^5^Department of Medical-Chemical Laboratory Analysis, Medical University of Vienna, Vienna, Austria

**Keywords:** prediction, diabetes, percutaneous coronary intervention, acute coronary syndrome, troponin

## Abstract

**Background:** Elevated levels of troponin are associated with future major adverse cardiac events (MACE). Data on the prognostic value of high sensitive troponin T (hs-TnT) compared to high sensitive troponin I (hs-TnI) in diabetic and non-diabetic patients are sparse.

**Methods:** We analyzed patients of a single-center registry undergoing coronary stenting between 2003 and 2006. As a primary endpoint we assessed MACE, a composite of cardiovascular death, nonfatal myocardial infarction and nonfatal stroke according to sex and diabetes status using log-rank. As a second endpoint, we assessed the prognostic impact of hs-TnT and hs-TnI on MACE, adjusting for known confounders using Cox regression analysis.

**Results:** Out of 818 investigated patients, 267 (32.6%) were female. Diabetes mellitus type 2 (T2DM) was diagnosed in 206 (25.2%) patients.

After a mean follow-up of 6.6 ± 3.7 years, MACE occurred in 235 (28.7%) patients. The primary endpoint components of cardiovascular death occurred in 115 (14.1%) patients, MI in 75 (9.2%), and ischemic stroke in 45 (5.5%). Outcomes differed significantly according to sex and diabetes status (*p* = 0.003). In descending order, MACE rates were as follows: female diabetic patients (40.8%), female non-diabetic patients (32.7%), male diabetic patients (28.9%), and male non-diabetic patients (24.8%). Additionally, females with diabetes were at higher risk of cardiovascular death compared to diabetic men (28 vs. 15%). Hs-TnI (HR 1.477 [95% CI 1.100–1.985]; *p* = 0.010) and hs-TnT (HR 1.615 [95%CI 1.111–2.348]; *p* = 0.012) above the 99th percentile were significantly associated with MACE. Both assays showed tendency toward association with MACE in all subgroups.

**Conclusion:** Diabetic patients, particularly females, with known coronary artery disease had a higher risk of subsequent MACE. Both, hs-TnI and hs-TnT significantly correlated with MACE.

## Introduction

Diabetes has been shown to be a major risk factor for adverse outcomes in patients with and without known coronary artery disease (CAD) (1–3). Also, female sex has been shown to be associated with worse outcomes compared to its male counterparts (4, 5).

Troponin T (TnT) and I (TnI) are unique markers of myocardial damage and necrosis, as both are expressed only in myocardial cells (6, 7). The use of troponin in the diagnosis of acute coronary syndrome (ACS) is well-established, and particularly the introduction of high-sensitivity troponin-assays has further improved the diagnosis of ACS (6–9). Adjacent to the clinical importance of troponin in the diagnosis of ACS, cardiac troponin is significantly related to subsequent major adverse cardiovascular events (MACE) (9–13). Similar findings have been shown for patients with stable CAD (SCAD), in which elevated levels of troponin were related to long-term MACE, although the mechanism of chronic troponin elevation in SCAD is still matter of debate (14). Chronic troponin elevations in SCAD patients are related to atherosclerotic plaque burden detected by CT scan and may reflect ongoing silent myocardial injury (15). Also, chronic troponin elevations may reflect silent rupture of non-calcified plaques and microembolization (16).

Estrogens have protective effects on the cardiovascular system (17), however this effect disappears in diabetic women. Diabetic women have been shown to be at higher risk of developing coronary heart disease (5) and are characterized by a worse cardiovascular risk profile (18). Especially elevated hs-TnI levels were shown to be significantly related to MACE, heart failure (HF), myocardial infarction (MI), and cardiovascular mortality in diabetic patients (19). However, there are also controversial data reporting a missing link between the levels of TnT and the prediction of future adverse outcomes in diabetic patients (20). To the best of our knowledge, to date there has been no direct comparison of hs-TnI and hs-TnT in giving prognostic information for diabetic men and women.

We therefore sought to assess the risk of MACE in (1) ACS and (2) SCAD patients according to gender and diabetes status undergoing percutaneous coronary intervention (PCI). Moreover, we wanted to compare the prognostic value of hs-TnT and hs-TnI with regards to clinical outcomes in diabetic and non-diabetic patients.

## Methods

### Study Population

We analyzed patients from a prospective single-center registry undergoing PCI and stenting between January 2003 and December 2006. Summarized, diagnosis of ACS or SCAD was established according to the guidelines effective at that time. ACS patients presented either with persistent ST-segment elevation myocardial infarction (STEMI) or non ST-elevation acute coronary syndromes (NSTE-ACS). SCAD was defined according to positive ischemia testing (treadmill examination, dobutamine stress echocardiography or single-photon-emission computed tomography). Stent type, antithrombotic regimen and secondary-prevention therapy were at the discretion of the treating interventionist. Laboratory results, clinical characteristics, cardiovascular risk factors, comorbidities, coronary morphology, and medication at hospital discharge were collected for all patients. Diabetes mellitus type 2 was diagnosed with the medical history of the patients, including antidiabetic medication.

Patients in whom PCI was not successful and those not residing in the Vienna metropolitan area were excluded from the present analysis. The present study complies with the Declaration of Helsinki of 1975 and was approved by the local ethics committee for human subjects of vienna (EK 10-046-VK_NZ). Written informed consent was obtained from all included subjects.

### Detection of Troponin

Blood samples for the detection of troponin I (Abbott Core Laboratories) and T (Roche) were collected right before coronary angiography, centrifuged immediately after collection and stored at −80°C until measurement. Hs-TnI and hs-TnT were measured using the commercially available assays of Abbott and Roche. The upper reference limit (99th centile) was determined by the manufacturers as 26 ng/L (hs-TnI) and 14 ng/L (hs-TnT).

### Clinical Outcomes

The endpoint of interest was MACE defined as a composite of cardiovascular death, non-fatal MI and non-fatal ischemic stroke. Regional and national databases were used to ensure complete follow-up for the entire study population.

Mortality data for all patients were obtained from the Statistics Austria Institute. The Statistics Austria Institute is an independent and non-profit federal public institution supporting scientific services. Data on recurrent myocardial infarction or ischemic stroke was obtained using the common Vienna regional hospital database system.

### Statistics

All continuous variables following normal distribution are expressed as mean ± standard deviation (*SD*) and all variables not following normal distribution were described as median (interquartile range [IQR]). Variables were tested for normal distribution using the Kolmogorov-Smirnov-Liliefors test. Categorical variables are expressed as numbers and percentages. Continuous variables were compared by either ANOVA or the Kruskal-Wallis-H-test, as appropriate. χ^2^-tests were performed for categorical variables.

Survival curves [all patients, ACS patients, stable coronary artery disease (SCAD) patients] were calculated using the Kaplan-Meier method and compared using the log-rank test. Cox proportional hazard models were applied in order to assess the prognostic impact of hs-TnT and hs-TnI on long-term MACE, adjusting for known confounders (age, sex, body-mass index, systolic blood pressure, cholesterol, HbA1c, smoking, heart failure, eGFR). We tested the proportional hazard assumption for all covariates using Schoenfeld residuals (overall test) and the scaled Schoenfeld residuals (variable-by-variable testing). According to the tests the proportional hazards assumption was not violated.

All statistical analyses were performed with SPSS 21.0 (SPSS Inc., Chicago, IL, USA), and R 3.4.0 (http://www.r-project.org). All statistical tests were 2-tailed, and a *p* < 0.05 was required for statistical significance.

## Results

### Patient Population

Out of 818 investigated patients, 267 (32.6%) were female. 395 (48.3%) patients presented with SCAD. Baseline characteristics, laboratory findings, procedural details and medication at hospital discharge are listed in Table 1, stratified according to sex and diabetes status. T2DM was diagnosed in 206 (25.2%) patients. Patients with T2DM were characterized by higher body mass index (BMI) and glucose profiles. In univariate correlation analyses hs-TnI levels were significantly associated with hs-TnT (*R* = 0.878, *p* < 0.001).

**Table 1:** Baseline characteristics of the study population, *N* = 818.

	**Female**		**Male**		
**Variables**	**Diabetes No**. **= 71**	**No diabetes No**. **= 196**	**Diabetes No**. **= 135**	**No diabetes No**. **= 416**	***p*****-value**
Hs-Troponin I (ng/L)	4,928 (9,862)	4,729 (10,432)	4,101 (9,526)	4,443 (10,751)	0.219
Hs-Troponin T (ng/L)	1,175 (2,101)	666 (1,517)	513 (1,239)	787 (1,755)	0.029
ACS vs. SCAD [%(n)]					0.047
SCAD	42% (30)	46% (91)	59% (79)	47% (195)	
ACS	58% (42)	54% (105)	41% (55)	53% (221)	
Age (years)	70.4 (9.9)	68.6 (13.3)	62.1 (10.4)	62.6 (12.4)	<0.001
BMI (kg/m^2^)	29.3 (5.3)	26.8 (4.7)	29.8 (4.7)	27.4 (4.1)	<0.001
Heart rate (beats per minute)	81.3 (18.6)	70.2 (16.0)	75.4 (17.7)	71.7 (16.5)	<0.001
Systolic blood pressure (mmHg)	142.9 (27.4)	138.1 (24.2)	138.7 (24.8)	140.1 (24.1)	0.621
Diastolic blood pressure (mmHg)	83.1 (13.7)	77.7 (13.2)	81.7 (15.0)	82.4 (15.0)	0.005
MACE [%(n)]	41% (29)	33% (64)	29% (39)	25% (103)	0.001
CV death [%(n)]	28% (20)	22% (43)	15% (20)	13% (55)	0.003
Stroke [%(n)]	8% (6)	6% (12)	7% (10)	4% (18)	0.357
MI [%(n)]	11% (8)	10% (19)	10% (14)	9% (38)	0.942
Total cholesterol (mg/dl)	186 (46)	196 (42)	182 (45)	189 (46)	0.057
HDL-cholesterol (mg/dl)	45 (12)	56 (21)	41 (13)	44 (13)	0.001
LDL-cholesterol (mg/dl)	103 (33)	115 (38)	100 (36)	114.9 (41)	0.004
Triglycerides (mg/dl)	181 (140)	128 (75)	195 (135)	150 (91)	0.001
Glucose (mg/dl)	191.7 (88.3)	122.9 (46.5)	161.5 (64.6)	117.2 (39.1)	0.001
HbA1c (%)	7.7 (1.5)	6.20 (1.6)	7.5 (1.1)	5.9 (0.7)	0.001
eGFR (ml/min/1,73 m^2^)	69 (31)	71 (27)	93 (24)	85 (26)	0.001
Creatinine (mg/dl)	1.2 (1.1)	0.9 (0.3)	1.0 (0.3)	1.1 (0.5)	0.001
Arterial hypertension	85.9% (61)	80.1% (157)	83.0% (112)	72.6% (302)	0.009
Hyperlipidaemia	66.2% (47)	68.4% (134)	77.0% (104)	70.9% (295)	0.280
Smoking	23.9% (17)	24.0% (47)	28.1% (28.1)	29.3% (122)	0.016
Prior myocardial infarction	31.0% (22)	18.4% (36)	32.6% (44)	21.2 (88)	0.005
Peripheral artery disease	16.9% (12)	5.6% (11)	11.9% (16)	4.6% (19)	<0.001
Central artery disease	5.6% (4)	5.1% (10)	9.6% (13)	4.8% (20)	0.205
Heart failure	19.7% (14)	10.2% (20)	17.0% (23)	9.1% (38)	0.010
Diabetes therapy [%(n)]					0.001
Insulin	15% (11)	.	10% (13)	.	
Oral antidiabetics	60% (43)		76% (102)		
Both	7% (5)	.	7% (10)	.	
Statin	91.3% (63)	84.2% (160)	87.8% (115)	87.4% (354)	0.462
Aspirin	98.4% (70)	99.5% (195)	97.0% (131)	99.8% (415)	0.024
P2Y12 inhibitor	94.4% (64)	95.4% (187)	93.4% (126)	94.7% (394)	0.400

*hs, high-sensitive; ACS, acute coronary syndrome; SCAD, stable coronary artery disease; BMI, body mass index; MACE, major adverse cardiac events; CV death, cardiovascular death; MI, myocardial infarction; HDL-cholesterol, high-density lipoprotein cholesterol; LDL-cholesterol, low-density lipoprotein cholesterol; CK, creatin kinase; GFR, glomerular filtration rate*.

Sex-specific differences between female and male diabetic patients:

Female diabetic patients were older, had higher hs-TnT levels and a higher occurrence of an ACS when compared to male diabetic patients. Additionally they were characterized by unfavorable glucose profiles when compared to male diabetic patients (e.g., higher glucose and HbA1c levels). As shown in Table 1, females with diabetes were at higher risk of MACE when compared to male diabetic patients (41 vs. 29%) and had a higher risk of cardiovascular death (28 vs. 15%).

### Outcome Analysis

#### Total Patient Cohort

**Figure 1:**
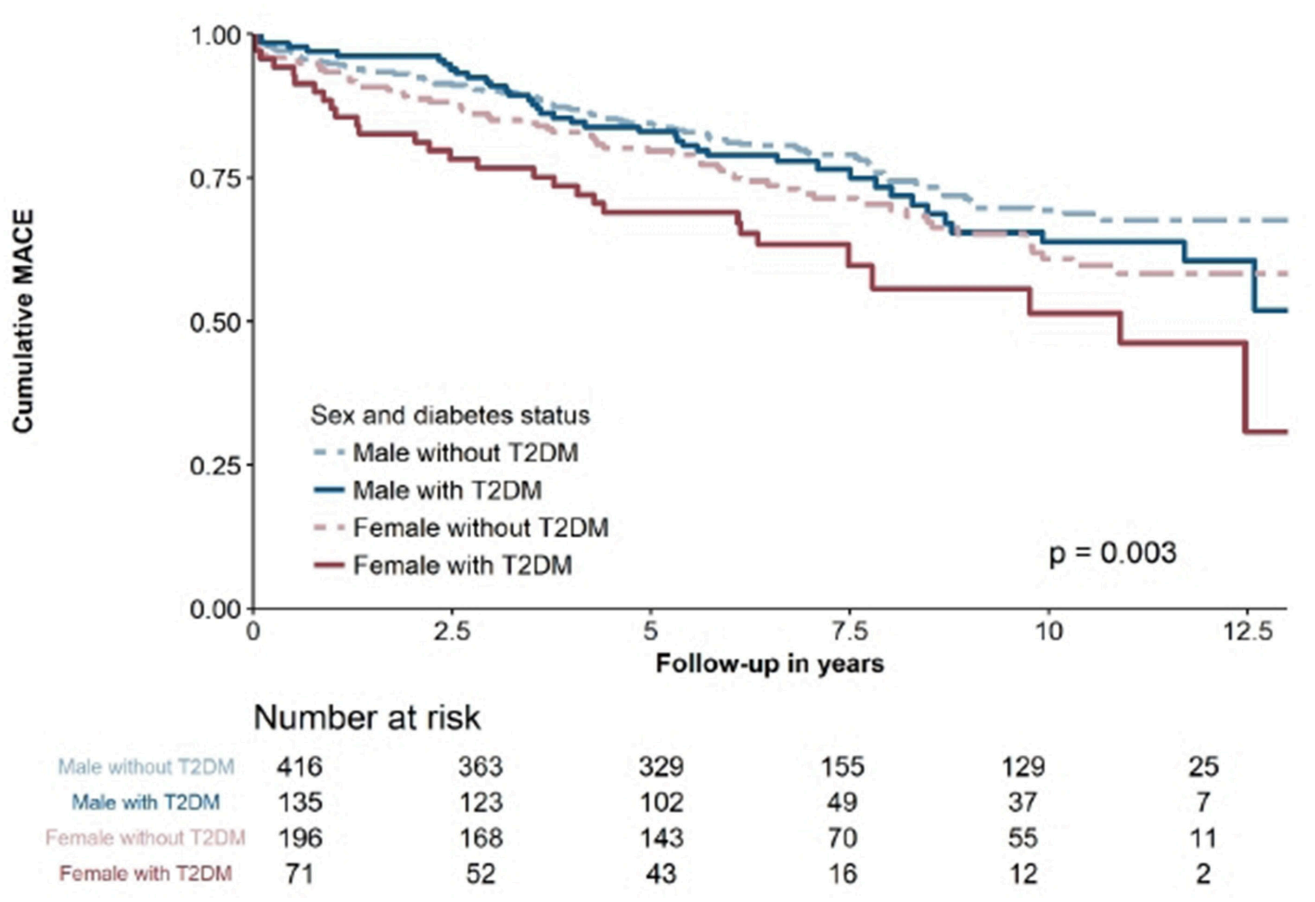
Kaplan-Maier survival curve for all patients stratified according to sex and diabetes status.

After a mean follow-up of 6.6 ± 3.7 years, MACE occurred in 235 (28.7%) patients. The primary endpoint components of cardiovascular death occurred in 115 (14.1%) patients, MI in 75 (9.2%), and ischemic stroke in 45 (5.5%). As depicted in Figure 1, outcomes differed significantly according to sex and diabetes status (*p* = 0.003). In descending order, MACE rates were as follows: female diabetic patients (40.8%), female non-diabetic patients (32.7%), male diabetic patients (28.9%), and male non-diabetic patients (24.8%). Hs-TnI (HR 1.477 [95% CI 1.100–1.985]; *p* = 0.010) and hs-TnT (HR 1.615 [95%CI 1.111–2.348]; *p* = 0.012) above the 99th percentile were significantly associated with MACE. Both hs-troponin assays showed a tendency toward association with future MACE in all subgroups, as shown in Table 2. Both models including all variables entered into the model are depicted in Table 3.

**Table 2:** Association using the 99th percentile of hs-TnT and hs-TnI with long-term MACE adjusted for confounders.

	**HR**	**95%CI**	***p*-value**
**ALL PATIENTS (818)**			
hs-TnI	1.477	1.100–1.985	**0.010**
hs-TnT	1.615	1.111–2.348	**0.012**
**FEMALE WITH T2DM (*****n*** **= 71)**			
hs-TnI	1.622	0.495–5.312	0.425
hs-TnT	2.055	0.502–8.418	0.317
**MALE WITH T2DM (*****n*** **= 135)**			
hs-TnI	2.737	0.989–5.853	0.062
hs-TnT	2.262	0.887–5.773	0.088
**FEMALE WITHOUT T2DM (*****n*** **= 196)**			
hs-TnI	1.561	0.879–2.773	0.129
hs-TnT	1.823	0.884–3.762	0.104
**MALE WITHOUT T2DM (*****n*** **= 416)**			
hs-TnI	1.203	0.779–1.857	0.404
hs-TnT	1.423	0.822–2.464	0.207

*HR, hazard ratio; CI, confidence interval; Hs, high-sensitive; T2DM, diabetes mellitus type 2. The bold values are the significant p-values*.

**Table 3:** Predictors of long-term composite MACE.

	**HR**	**95% CI**	***p*-value**
**FINAL MODEL**			
hs-TnI	1.477	1.100–1.985	0.010
Age	1.028	1.010–1.046	0.003
Sex	0.987	0.744–1.308	0.926
BMI	0.992	0.958–1.027	0.654
HbA1c	1.179	0.988–1.407	0.067
Systolic blood pressure	1.004	0.998–1.011	0.192
eGFR	0.988	0.980–0.996	0.003
Total cholesterol	0.998	0.994–1.001	0.147
Smoking	0.836	0.661–1.059	0.138
Heart failure	1.910	1.330–2.742	<0.001
hs-TnT	1.615	1.111–2.348	0.012
Age	1.026	1.008–1.045	0.005
Sex	1.024	0.770–1.362	0.871
BMI	0.992	0.958–1.027	0.635
HbA1c	1.135	0.965–1.333	0.125
Systolic blood pressure	1.004	0.997–1.010	0.258
eGFR	0.989	0.981–0.997	0.009
Total cholesterol	0.998	0.995–1.001	0.234
Smoking	0.844	0.663–1.076	0.171
Heart failure	1.792	1.241–2.586	0.002

*Legend: HR, hazard ratio; CI, confidence interval; BMI, body-mass index; eGFR, estimated glomerular filtration rate*.

#### ACS Patients

**Figure 2:**
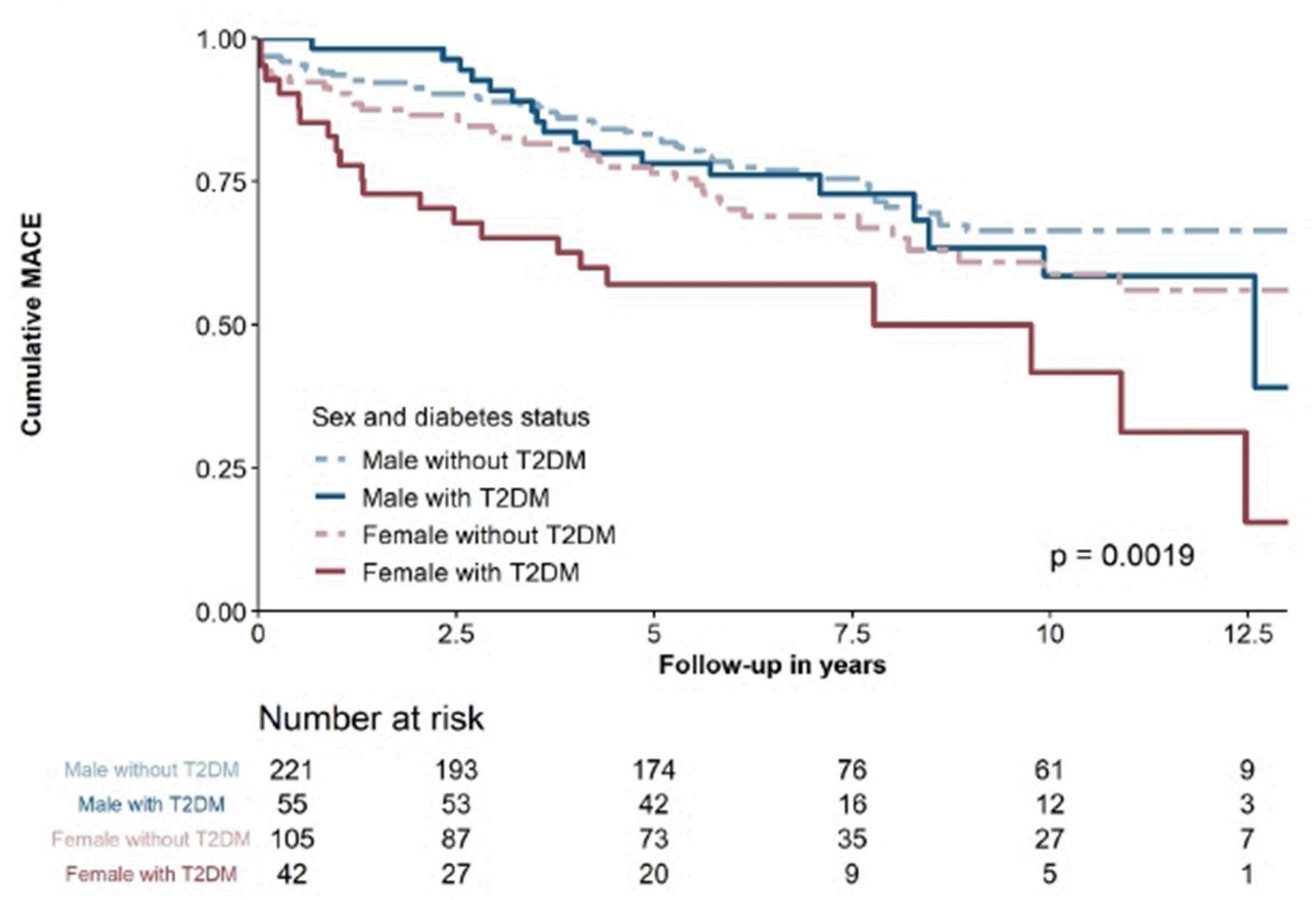
kaplan-Maier survival curve of ACS-patients stratified according to sex and diabetes status.

In the ACS cohort, 136 (16.6%) patients suffered from MACE. From these, 65 (7.9%) were classified as cardiovascular death, 47 (5.7%) as non-fatal MI and 24 (2.9%) as non-fatal stroke or TIA. As shown in Figure 2, outcomes differed significantly according to sex and diabetes status (*p* = 0.0019). Neither, hs-TnI (HR 1.001 per 100 ng/l increase [95%CI 1.000–1.002]; *p* = 0.100) nor hs-TnT (HR 1.007 per 100 ng/l increase [95%CI 1.000–1.015]; *p* = 0.066) were significantly associated with MACE.

#### SCAD Patients

**Figure 3:**
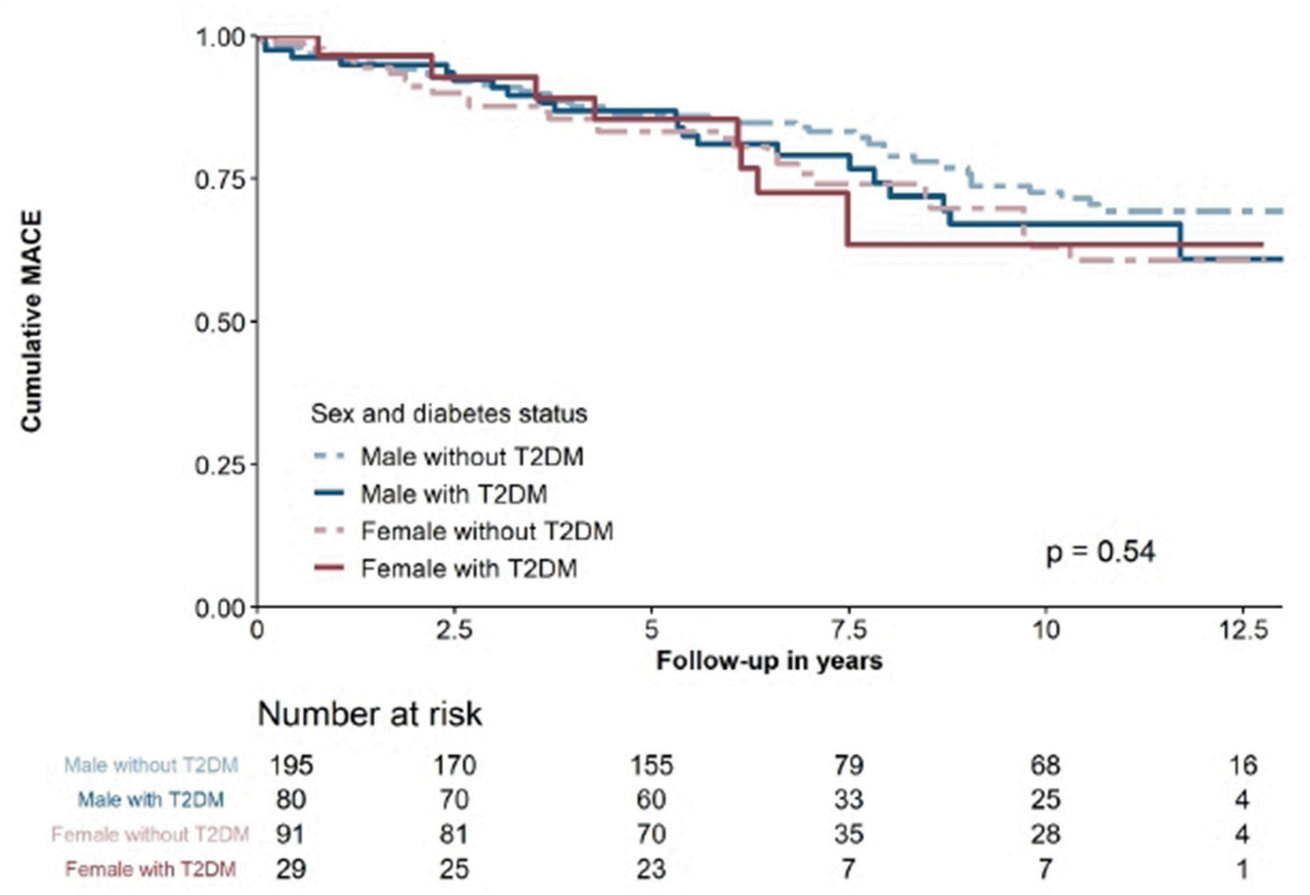
Kaplan-Maier survival curve of SCAD patients stratified accordong to sex and diabetes status.

In the SCAD cohort, 99 (12.1%) patients suffered from MACE, attributable to 50 (6.1%) with cardiovascular death, 28 (3.4%) with non-fatal MI and 21 (2.6%) with non-fatal stroke. As demonstrated in Figure 3, there were no significant differences according to sex and diabetes status (*p* = 0.54). In SCAD patients, neither hs-TnI (HR 1.001 per 100 ng/l increase [95%CI 0.994–1.007]; *p* = 0.889) nor hs-TnT (HR 0.999 per 100ng/l increase [95%CI 0.920–1.067]; *p* = 0.804) were significantly associated with MACE.

## Discussion

In our study cohort, diabetic patients with CAD undergoing PCI, particularly females, had a higher risk of subsequent MACE over the course of a median of 6.6 years compared to their non-diabetic counterparts. Especially female diabetic patients were at higher risk of developing the single end point of cardiovascular death when compared to diabetic men.

Moreover, to the best of our knowledge, this is the first study to investigate potential prognostic differences of two distinct troponin assays comparing patients with and without T2DM, hs-TnT, and hs-TnI. Both assays were significantly associated with MACE and showed a tendency toward association with MACE in all subgroups, although did not reach statistical significance, assumedly due to the small sample size.

Diabetes has been shown to be a major risk factor for adverse outcomes in patients with and without known coronary artery disease (1–3). Also, the female sex has been shown to be associated with worse outcomes in cohorts with either high risk for CAD or overt CAD compared to its male counterparts (4, 5). In consequence, in our cohort female diabetic patients were most prone to suffer from subsequent cardiovascular events compared to the other subgroups. Similar results have been published previously by Peters and Huxley, who demonstrated a 44% greater risk of diabetic women to develop CAD and 46% greater risk for cardiovascular death compared to diabetic men (5). Also, a recently published register-based cohort study presented that women with type 1 diabetes are at higher risk of developing CAD and have a higher mortality rate when compared to male patients with type 1 diabetes. Therefore, Rawshani et al. stated that it is important to reduce the risk profile in type 1 diabetic women, including smoking cessation, glycaemic control, and control of blood pressure and lipid parameters in order to extend life expectancy and to reduce the sex-specific differences in major adverse cardiac events in type 1 diabetic patients (21). Thus, our results also showed that women with T2DM are at higher risk of MACE and cardiovascular death when compared to men with T2DM. Several biological and healthcare system-related factors might explain these sex-specific differences concerning the adverse outcomes in female patients. In earlier studies, post-menopausal women in particular have been shown to have an increased risk of cardiovascular mortality (22). This is most likely due to the loss of estrogen-driven athero-protective effects, an impact which is even more pronounced in presence of T2DM (17). Additionally it has been demonstrated that diabetic women have an increase in endothelin-1 and oxidative stress, known factors related to enhanced atherosclerosis and thrombotic risk. Diminished collateral vessel development has also been described in women with T2DM (23). Moreover, the incidence of atypical symptoms of CAD is higher in women compared to men, leading to delays in diagnosis and treatment of CAD, known effectors of adverse outcomes, particularly in the case of ACS (24, 25). Furthermore, during coronary intervention women have higher risk of bleeding complications (26, 27). Also, poorer risk factor control, i.e., in case of arterial hypertension, compared to male patients has been reported (28). In patients with acute chest pain, TnI or TnT are established markers for diagnosing ACS (8). Both molecules are components of the contractile apparatus of cardiomyocytes with unique functions. Traditionally, TnT and TnI have been considered markers of myocardial necrosis, but recent studies have also shown small levels circulating in patients with SCAD (29). Studies suggest that factors influencing chronic troponin elevation may differ between hs-TnT and hs-TnI and describe only a moderate correlation between these assays (30). In order to further enhance early diagnosis of ACS, high-sensitivity assays have been introduced, even allowing detection of very low levels of circulating troponin (9, 31). Further, detectable levels of these hs-Tn assays have been shown to be associated with adverse outcomes not only in ACS but also in SCAD (30). Albeit being associated with adverse outcomes, the introduction of hs-assays in ACS populations have so far not led to an additional reduction of adverse outcomes when compared with the contemporary assays (13).

Little is known about potential differences in chronic release and degradation patterns with possible prognostic impact, particularly in the field of diabetes. To the best to our knowledge, we are the first to compare possible sex-specific differences of hs-TnT and hs-TnI on prognostic impact in patients with or without diabetes and known CAD. We demonstrate that although overall not significant, both assays at least showed a tendency toward association with MACE. Recently, Everett et al. investigated the prognostic impact of cardiovascular outcomes in diabetic women without CAD and could demonstrate that hs-TnT is effective in providing prognostic information on CVD events (10). Other studies which investigated diabetic patients without CAD also showed that hs-TnI is a robust predictor of clinical outcomes and demonstrated that increased levels of hs-TnI are related to an increased risk of MACE, heart failure and cardiovascular mortality in diabetic patients (11). However, as we only included patients with known CAD undergoing PCI, the comparability is limited.

## Limitations

The present investigation should be interpreted with the following limitations in mind: in order to reduce heterogeneity in our cohort these results were derived from a single-center patient population undergoing PCI. Therefore, our results cannot be extrapolated to patients with CAD treated medically or with failed PCI and those who underwent surgical revascularization. Furthermore, both assays were investigated at a single time-point during index hospitalization before stent implantation. Temporal changes after PCI and, moreover, initiation of antiplatelet therapy might have influenced our results. Finally, the diagnosis of diabetes was evaluated using the medical history and not by oral glucose tolerance test and no data on diabetes duration are available.

## Conclusion

Diabetic patients, particularly female patients, with known coronary artery disease had a higher risk for subsequent MACE over the course of a median of 6.6 years compared to their non-diabetic counterparts. Overall both, hs-TnI and hs-TnT significantly correlated with MACE. Especially the results of the present study which showed that women with T2DM are at higher risk of MACE and cardiovascular death when compared to men with T2DM, have clinical relevance. Therefore, especially the high risk population of T2DM diabetic women should be screened more commonly and the risk profiles, including lipid parameters, glycaemic control or arterial hypertension should be treated more strictly and in accordance with the guidelines.

## Author Contributions

ML, MT, and SF performed statistical analysis and calculations. ML and MT wrote the manuscript. All authors reviewed and edited the manuscript.

### Conflict of Interest Statement

The authors declare that the research was conducted in the absence of any commercial or financial relationships that could be construed as a potential conflict of interest.
